# Al–Bi_2_Se_3_–Al Nanoribbon Josephson Junctions with Fabry–Pérot Interference: Implications for Phase-Coherent Topological Insulator-Based Superconducting Devices

**DOI:** 10.1021/acsanm.6c01243

**Published:** 2026-07-01

**Authors:** Kiryl Niherysh, Nermin Trnjanin, Ananthu P. Surendran, Gunta Kunakova, Xavier Palermo, Domenico Montemurro, Jana Andzane, Donats Erts, Dmitry S. Golubev, Samuel Lara-Avila, Floriana Lombardi, Thilo Bauch

**Affiliations:** † Quantum Device Physics Laboratory, Department of Microtechnology and Nanoscience, Chalmers University of Technology, Göteborg SE-41296, Sweden; ‡ Institute of Chemical Physics, Faculty of Science and Technology, University of Latvia, Riga LV-1586, Latvia; § Dipartimento di Fisica “Ettore Pancini”, Universitá Degli Studi di Napoli Federico II, Napoli I-80125, Italy; ∥ QTF Centre of Excellence, Department of Applied Physics, Aalto University, Aalto FI-00076, Finland

**Keywords:** Bi_2_Se_3_ nanoribbons, Josephson junctions, Fabry−Pérot interference, transverse quantization, quasi-ballistic transport.

## Abstract

We investigate phase-coherent quantum transport in nanoscale Al–Bi_2_Se_3_–Al nanoribbon Josephson junctions by combining normal-state conductance spectroscopy with a junction-length-dependent study of Josephson transport. Differential conductance maps versus bias and gate voltage reveal Fabry–Pérot interference, whose periodicity matches the nanoribbon width, consistent with transverse quantization and quasi-ballistic surface-state trajectories in 430 nm wide devices. A systematic investigation of the characteristic voltage *I*_*c*_ *R*_*n*_ as a function of junction length *L* exhibits a clear plateau for *L* ≤ 500 nm, indicative of a short ballistic contribution to the Josephson transport, and decreases for longer junctions as diffusive transport dominates. Together, Fabry–Pérot interference and Josephson transport measurements provide complementary, channel-selective evidence for quasi-ballistic surface-state transport persisting over several hundred nanometers in hybrid topological insulator nanostructures. These results demonstrate the potential of Bi_2_Se_3_ nanoribbon Josephson junctions as a nanoscale platform for phase-coherent superconducting electronics, topological quantum computing architectures, and topological spintronic devices.

## Graphical abstract

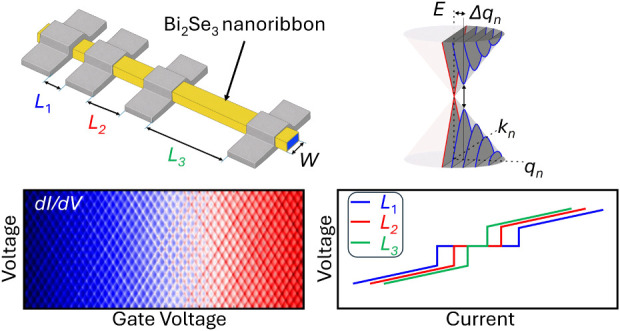

## Introduction

Understanding how quasi-ballistic transport manifests in the topological surface states of hybrid topological insulator (TI)–superconductor devices is crucial for the development of architectures for topological quantum computing.^1,2^ Moreover, the inherent characteristic of spin-momentum locking within TIs opens up a promising avenue for efficient spin manipulation and detection.^3–6^ In ballistic mesoscopic conductors, coherent multiple reflections between interfaces can produce Fabry–Pérot (FP) interference, resulting in oscillatory conductance and resonant features in the supercurrent.^7,8^ Such interference effects have been widely observed in systems exhibiting quasi-ballistic phase-coherent transport, including carbon nanotubes, quantum Hall edge states, semiconducting nanowires, graphene nanoribbons, and topological insulator devices.^9–19^ The underlying mechanism is constructive interference between electron waves reflected at two interfaces, resulting in resonant transmission whenever *k*_*F*_ *L* = *πn*, with *k*_*F*_ the Fermi wave vector, *L* the distance between the interfaces, and *n* a nonzero integer.^9^ These FP resonances typically manifest as periodic oscillations in device conductance as a function of chemical potential^9,10,12^ and can be interpreted as transport through quasi-bound states formed by finite-size quantization between  interfaces.

In conventional materials with two-dimensional (2D) device geometries, angular averaging over the various directions of electron propagation typically leads to a vanishing interference pattern.^20^ In contrast, Dirac materials, such as graphene and TIs, exhibit Klein tunneling^21^ which gives rise to a subset of highly transmissive propagation trajectories (modes). As a result, interference effects can remain robust and experimentally observable even in 2D geometries.^11,19,22–25^

In addition to the FP resonances dictated by the device length, additional resonances can emerge with a periodicity determined by the sample width, originating from transverse quantization.^23,24,26,27^ In contrast, universal conductance fluctuations (UCF) produce quasi-periodic, sample-specific patterns whose characteristic scales bear no relation to the device dimensions. Distinguishing FP interference from UCF is therefore essential, as they correspond to fundamentally different transport regimes.

A complementary probe of quasi-ballistic surface transport can be achieved through the distinct length dependence of the Josephson effect. In a TI Josephson junction, increasing the junction length *L* effectively filters the contributing transport channels: the diffusive bulk contribution to the supercurrent decays rapidly with *L*, while ballistic surface trajectories can sustain an approximately length-independent Josephson current over distances up to the mean free path, which can reach several hundred nanometers. Consequently, analysis of the *I*_*c*_ *R*_*n*_ product, together with other Josephson transport properties, can reveal a regime in which the supercurrent is predominantly carried by ballistic surface states.

Our study combines two complementary data sets obtained from two distinct Bi_2_Se_3_ nanoribbons: (i) two narrow junctions (devices **C1** and **C2**) on nanoribbon **NR1**, used to analyze FP-like interference within the same ribbon; and (ii) a series of Josephson junctions with varying lengths on nanoribbon **NR2**, used to examine the characteristic voltage *I*_*c*_ *R*_*n*_ as a function of junction length, where *I*_*c*_ is the critical current and *R*_*n*_ is the junction resistance. Together, these measurements indicate quasi-ballistic surface state transport over length scales up to ∼500 nm, evidenced by Fabry–Pérot resonances in 430 nm wide junctions and by saturation of the *I*_*c*_ *R*_*n*_ product for junction lengths *L* ≤ 500 nm.

## Fabry–Pérot Resonance in TI-Based Nanoribbons

Here, we analyze how FP resonances evolve with chemical potential and applied bias voltage. We begin by considering the zero-bias conductance. The system consists of a TI nanoribbon-based junction of length *L*, corresponding to the distance between the Al electrodes. The lateral dimensions of the device are defined by the width *W* and thickness *t* of the TI nanoribbon, as shown in Figure 1a.

**Figure 1:**
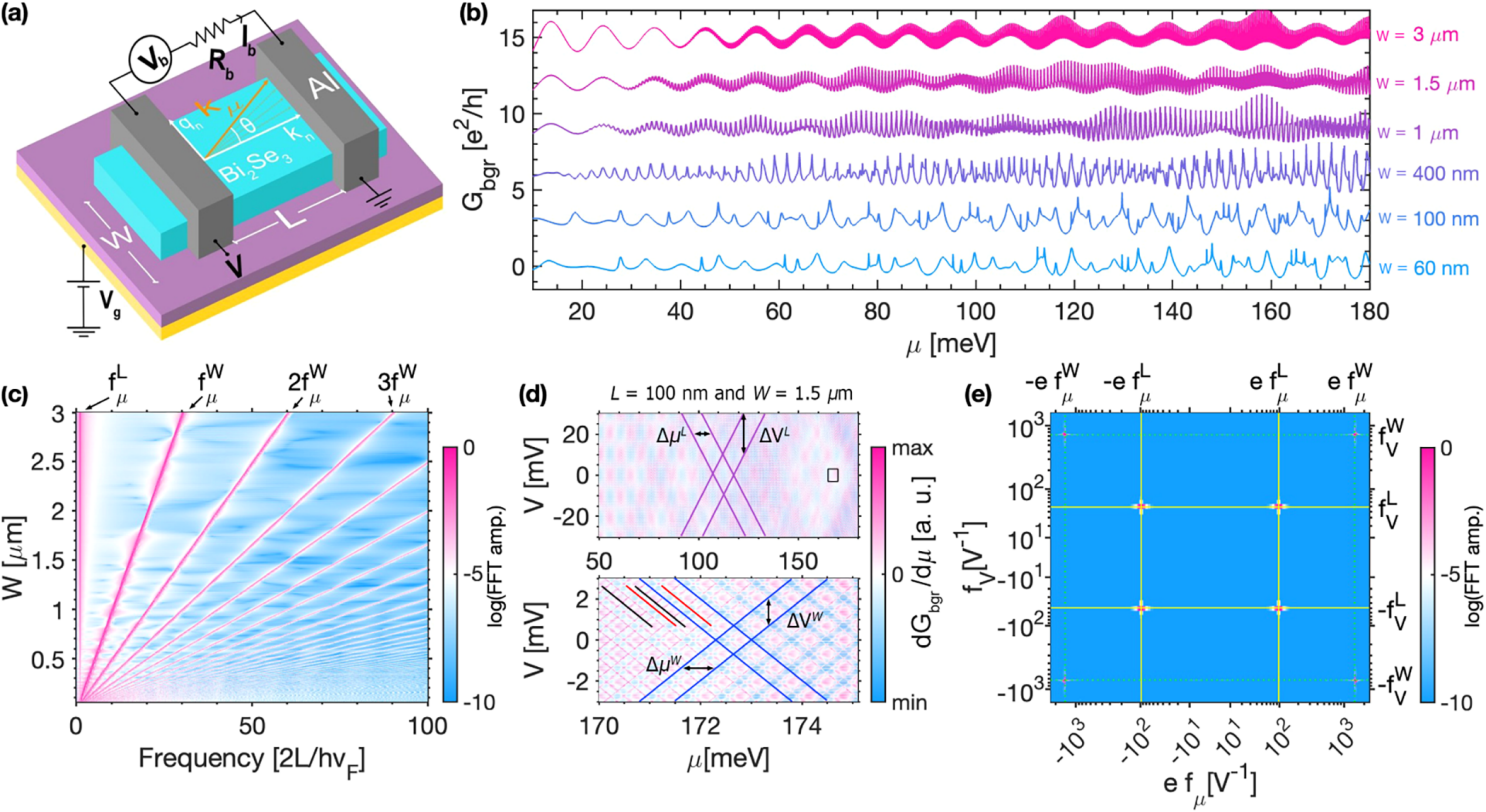
(a) Schematic of the device geometry. (b) Numerically calculated conductance as a function of chemical potential for a device with *L* = 100 nm and widths ranging from 60 nm to 3 μm. Linear backgrounds are subtracted, and curves are offset for clarity. (c) Fourier transforms of the conductance traces shown in (b) vs chemical potential for different widths. (d) Fabry–Pérot oscillations of the conductance as a function of chemical potential and source-drain bias for *L* = 100 nm and *W* = 1.5 μm (upper panel). Purple lines indicate a Fabry–Pérot diamond associated with resonances along the junction length. The spacing between adjacent parallel lines along the chemical potential direction is Δμ*^L^*, while the distance along the bias voltage axis corresponds to *e*Δ*V^L^* = 2Δμ*^L^*. The lower panel shows a zoom of the boxed area, highlighting Fabry–Pérot diamonds arising from transverse quantization with periodicities Δμ*^W^* and *e*Δ*V^W^* = 2Δμ*^W^*. Additional red and black guidelines in the lower panel highlight different families of parallel resonance lines with the same underlying periodicity but shifted positions along the chemical potential axis. (e) Two-dimensional Fourier transform of the Fabry–Pérot pattern shown in the upper panel of (d). Solid yellow and dashed green lines indicate the characteristic frequencies $f_{\mu }^{L}$ and $f_{\mu }^{W}$ , respectively.

For each transport mode in the topological surface states (TSS), the momentum *k* can be decomposed into transverse and longitudinal components *q*_*n*_ and *k*_*n*_, respectively. These components define the associated mode trajectory angle *θ* = tan^–1^(*k*_*n*_/*q*_*n*_) (see Figure 1a). The transverse momenta in the TI junction are quantized, forming sub-bands according to *q*_*n*_ = 2*π*(*n* + 1/2)/*P*, where *n* = 0, ±1, ±2, ··· and *P* = 2­(*W + t*) is the perimeter of the nanoribbon.^28,29^ For simplicity, we consider the case where *W* ≫ *t*, such that *q*_*n*_ ≃ (*n* + 1/2)­π/*W*. For a given energy *μ* = *ϵ* – *ϵ*_D_, measured relative to the Dirac point ϵ_*D*_, and assuming a Fermi velocity *v*_*F*_, we calculate the transmission probability for each transport mode in the TSS. In the limit of a large chemical potential mismatch between the uncovered TI region and the TI region covered by Al, the transmission is given by:^30^

1 $$\tau_{n}\left(\mu \right)=\frac{k_{n}^{2}}{k_{n}^{2}\cos^{2}\left(k_{n}L\right)+{\left(\mu /\hbar v_{F}\right)}^{2}\sin^{2}\left(k_{n}L\right)}$$

with $k_{n}=\sqrt{{\left(\mu /\hbar v_{F}\right)}^{2}-q_{n}^{2}}$ . Using the Landauer formula, the normal-state (zero-bias) conductance of the device can be expressed as $G\left(\mu \right)=G_{0}\underset{n}{\sum }\tau_{n}\left(\mu \right)$ , where *G*_0_ = *e* ^2^/*h*, *h* is the Planck’s constant, and *e* is the elementary charge.

We calculated the zero-bias conductance of a topological insulator junction as a function of the chemical potential μ for a fixed junction length *L* = 100 nm and a typical Fermi velocity *v*_*F*_ = 5 × 10^5^ m/s,^31^ while varying the junction width *W*. The results of these calculations are shown in Figure 1b, after removing the linear backgrounds from each curve for clarity (denoted as *G*_*bgr*_).

For wide devices, at low chemical potentials (near the Dirac point), we clearly observe sinusoidal conductance oscillations with a period given by Δ*μ* ^*L*^ = ℏ*v*_*F*_Δ*k*_*F*_ = *hv*_*F*_/2*L*, with Δ*k*_*F*_ = π/*L*. These oscillations arise from finite-size quantization along the length of the junction. At higher chemical potentials, faster oscillations become increasingly prominent. Notably, the oscillations associated with width quantization do not exhibit a sinusoidal form; instead, they appear as sharp, spike-like features. As we discuss below, these fast oscillations are characterized by a period *Δμ* ^*W*^ ≃ *hv*_F_/2*W*, originating from finite-size quantization along the perimeter of the nanoribbon (∼2*W*). This reflects the energy spacing between transverse 1D sub-bands.^28,29,32^

For junction widths approaching the junction length, conductance oscillations are still present. However, the superposition of conductance oscillations with similar periods (Δ*μ^W^* ≃ Δ*μ* ^*L*^) makes it challenging to identify periodic oscillations. Figure 1c shows the Fourier transforms of the conductance oscillations as a function of chemical potential for various junction widths. The low-frequency peak (vertical pink line) corresponds to the device length, given by $f_{\mu }^{L}=1/\Delta \mu^{L}=2L/hv_{F}$ , which is independent of the junction width. At higher frequencies, we identify a peak that varies linearly with the width $f_{\mu }^{W}=1/\Delta \mu^{W}=2W/hv_{F}$ . In addition, higher-order harmonics appear at frequencies $m\cdot f_{\mu }^{W}$ , where *m* is an integer greater than one. These harmonics originate from the strongly nonsinusoidal, nearly spike-like oscillations visible in Figure 1b.

Up to this point, we have focused only on the zero-bias conductance of the devices. Following *Oksanen et al.,* ^27^ we numerically calculate the differential conductance of a device with *L* = 100 nm and *W* = 1.5 μm at finite source-drain voltage *V* using the Landauer expression for the current flowing through the device:

2 $$I_{b}\left(\mu_{0},V\right)=\left(2e/h\right)\int_{\epsilon_{l}}^{\epsilon_{r}}\tau \left(\mu \right)d\mu$$

Here, *τ*(*μ*) is the transmission probability as a function of energy, given by eq 1. The electrochemical potentials of the left and right contacts are defined as *ϵ*_*l*_ = *μ*_0_ + *αeV* and *ϵ*_*r*_ = *μ*_0_ – (1 – *α*)*eV* , with *μ_0_* the chemical potential measured relative to the Dirac point and α describing the asymmetry of the applied bias drop across the junction. For symmetric contacts, α = 1/2, corresponding to an equal voltage drop at both interfaces (see Supporting Information 1). Applying a finite source-drain bias additionally induces a shift of the chemical potential relative to the Dirac point by Δμ = (α – 1/2)*eV*. The resulting conductance derivative *dG*_*bgr*_/*dμ* as a function of source-drain bias *V* and chemical potential *μ* is shown in Figure 1d. On large energy scales, we observe the characteristic Fabry–Pérot diamond pattern with a periodicity Δμ^*L*^ = *hv*_*F*_/2*L* ≃ 10.3 meV, associated with interference along the length of the junction (upper panel of Figure 1d). The corresponding periodicity along the source-drain voltage direction is given by Δ*V* ^*L*^ = 2Δμ^*L*^/*e*, where the factor of 2 is related to the assumption of symmetric contacts. On smaller energy scales (lower panel of Figure 1d), additional oscillations with periodicity Δμ*^W^* ≃ *hv*_*F*_/2*W* ≃ 0.69 meV are observed, originating from transverse quantization. The corresponding period along the source-drain voltage direction is similarly given by Δ*V* ^*W*^ = 2Δμ^*W*^/*e*. To identify the dominant frequency components, we compute the two-dimensional Fourier transform (2D-FFT) of the full conductance map (Figure 1e). This analysis reveals two frequencies $f_{\mu }^{L}$ and $f_{\mu }^{W}$ along the *f*_μ_-axis.^27^ Along the *f*_*V*_-axis, the peaks appear at frequencies $f_{V}^{L}=ef_{\mu ,L}/2$ and $f_{V}^{W}=ef_{\mu }^{W}/2$ .

## Results and Discussion

The Bi_2_Se_3_ nanoribbons were grown by physical vapor deposition, as described elsewhere.^31,33,34^ The growth was carried out in a horizontal single-zone quartz tube furnace on glass substrates. Owing to the rhombohedral *R*3̅*m* crystal structure (Figure 2a), together with the applied temperature and carrier-gas flow gradients, the nanoribbons preferentially grow laterally along the in-plane [110] direction, while the van der Waals layers remain stacked along the out-of-plane *c*-axis [001].^31^

**Figure 2:**
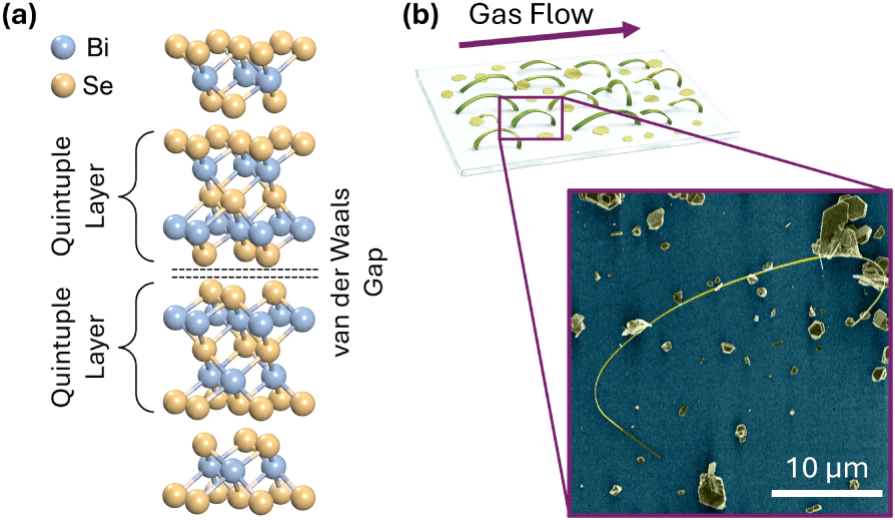
(a) Layered crystal structure of Bi_2_Se_3_, where each charge-neutral quintuple layer (QL) consists of five covalently bonded atomic sheets arranged in the sequence Se–Bi–Se–Bi–Se and separated by van der Waals gaps. (b) Schematic illustration of the as-grown Bi_2_Se_3_ nanoribbons on a glass substrate aligned along the carrier-gas flow direction (top panel), and a false-colored SEM image of an individual free-standing Bi_2_Se_3_ nanoribbon (bottom panel).

The as-grown free-standing stoichiometric nanoribbons (Figure 2b) were transferred onto commercially available Si substrates covered with a 300 nm thick thermally grown SiO_2_ layer using a dry flip-chip transfer technique. The substrates were prepatterned with alignment markers, and their backsides were coated with a Ti/Au layer to enable electrostatic back-gating. After transfer, the nanoribbons were characterized by optical and atomic force microscopy to identify suitable candidates for device nanofabrication.

The Josephson junction electrodes were defined by electron-beam lithography, followed by electron-beam evaporation of a Pt­(3 nm)/Al (80–120 nm) bilayer. Before metal evaporation, the native oxide from the Bi_2_Se_3_ nanoribbons was removed with the help of Ar^+^-ion milling.^33,34^

Such nanoribbons typically exhibit two-dimensional carrier densities on the order of 10^13^ cm^–2^, as determined from magnetotransport measurements in our earlier work,^35^ indicating contributions from both topological surface states and bulk states to the electrical transport. Unless stated otherwise, all measurements discussed below were carried out in a dilution refrigerator equipped with RC filters at 4 K and copper powder filters at the base temperature of 20 mK.

The devices originate from two nanoribbons: **NR1** (devices **C1** and **C2**) and **NR2** (a length-series used to study *I*_*c*_ *R*_*n*_ product). The experimental setup used for electrical characterization, together with a false-colored SEM image of junctions **C1** and **C2** fabricated on the same Bi_2_Se_3_ nanoribbon **NR1** (*W* ≃ 430 nm, *t* ≃ 16 nm), is shown in Figure 3a. The SEM image of device **C3,** realized on nanoribbon **NR2** to study the length dependence of the Josephson effect, is shown in Figure 3b. The high quality and reproducibility of the metallic Al contacts are reflected in the rather reproducible proximity gap Δ′ ≃159 ± 5 μeV, measured on device **C3** and extracted from the gap and subgap features in *dV*/*dI*_*b*_ spectra as a function of bias voltage (see Figure  S3 in Supporting Information 2).

To investigate the normal-state transport properties of the devices **C1** and **C2**, an out-of-plane magnetic field of 30 mT was applied to quench superconductivity in the Al electrodes. The differential conductance matrix of the devices as a function of bias voltage *V* and gate voltage *V*_*g*_ was obtained by first measuring the current–voltage characteristics (IVCs) at each gate voltage and then numerically differentiating the resulting curves to extract *G* = *dI*_*b*_/*dV*. To enhance the visibility of fine features, we subtracted an averaged conductance trace *G*_*aver*_(*V*), calculated over all gate voltages. For devices **C1** and **C2**, *G*_*aver*_(*V*) ≈ 120 and 80 *e* ^2^/*h*, respectively. This was followed by a linear background removal along the gate-voltage axis. The resulting background-corrected differential conductance maps *G*_*bgr*_ of the TI junctions in their normal state are presented in Figures 4a and d. For completeness, the unprocessed (raw) conductance maps for the measured devices are provided in Supporting Information 3 (see Figure S4).

To further analyze the data, we follow the approach described in ref [^27^] and compute the derivative of the conductance map with respect to the gate voltage, *dG*_*bgr*_/*dV*_*g*_, in order to enhance the visibility of diagonal features (indicated by lines) in the conductance maps, as shown in Figures 4b and e. We then apply a two-dimensional fast Fourier transform to determine the characteristic periods of these oscillations. In the 2D-FFT map shown in Figure 4c (device **C1**), we observe four distinct peaks at frequencies of ≃±204.00 V^–1^ along the *f*_*V*_ direction and ≃±0.075  V^–1^ along the *f_Vg_* direction (see the intersections of the horizontal and vertical dashed magenta lines). The frequency extracted along the source-drain bias direction is close to the expected value $f_{V}^{W}=208\;V^{-1}$ , which is associated with 1D sub-band quantization (due to momentum confinement around the nanoribbon perimeter). The 2D-FFT map shown in Figure 4f (device **C2**) exhibits four peaks at frequencies close to those observed in device **C1** (≃±216 V^–1^ along the *f*_*V*_ and ≃±0.095 V^–1^ along the *f_V_*_*g*_ direction, respectively). While two of these peaks are less intense and therefore less apparent in the raw FFT map, all frequency components predicted by the Fabry–Pérot model are present in both devices. The reduced intensity of some peaks likely arises from several factors, including nonideal contact transparency and residual bulk disorder, which can lead to a partial superposition of the FP diamond-like interference pattern with more irregular features resulting from universal conductance fluctuations. From the measured peak position $f_{V}^{W}$ , we extract a sub-band energy spacing of $\Delta \mu^{W}=e/2f_{V}^{W}=2.40\;\mathrm{meV}$ . The reproducibility of the observed frequencies within the same nanoribbon further supports the FP interpretation of the observed conductance oscillations.

The extracted transverse FP periodicity is compatible with two possible confinement scenarios. Besides simple width quantization *q*_*n*_ = *πn*/*W*, the observed frequencies are also consistent with quantization of circumferential topological surface state modes extending around the nanoribbon perimeter *q*_*n*_ = 2π­(*n* + 1/2)/*P*, where *P* is the ribbon perimeter. In topological insulator nanoribbons, such circumferential modes are expected to exhibit a gate-independent sub-band spacing Δ*q* = 2π/*P*, even in the presence of a nonuniform electrostatic potential around the ribbon circumference, owing to the Klein-tunneling nature of the Dirac surface states.^36^ Consequently, the observed periodicity can be interpreted within either confinement scenario, and the present measurements do not unambiguously identify which ribbon surfaces dominate the transport. However, given the nanoribbon geometry and the known presence of circumferential topological surface states in TI nanoribbons,^36^ we consider transport involving both the top and bottom surfaces to be the more likely scenario. Importantly, both interpretations remain fully consistent with the observation of phase-coherent quasi-ballistic FP interference.

**Figure 3:**
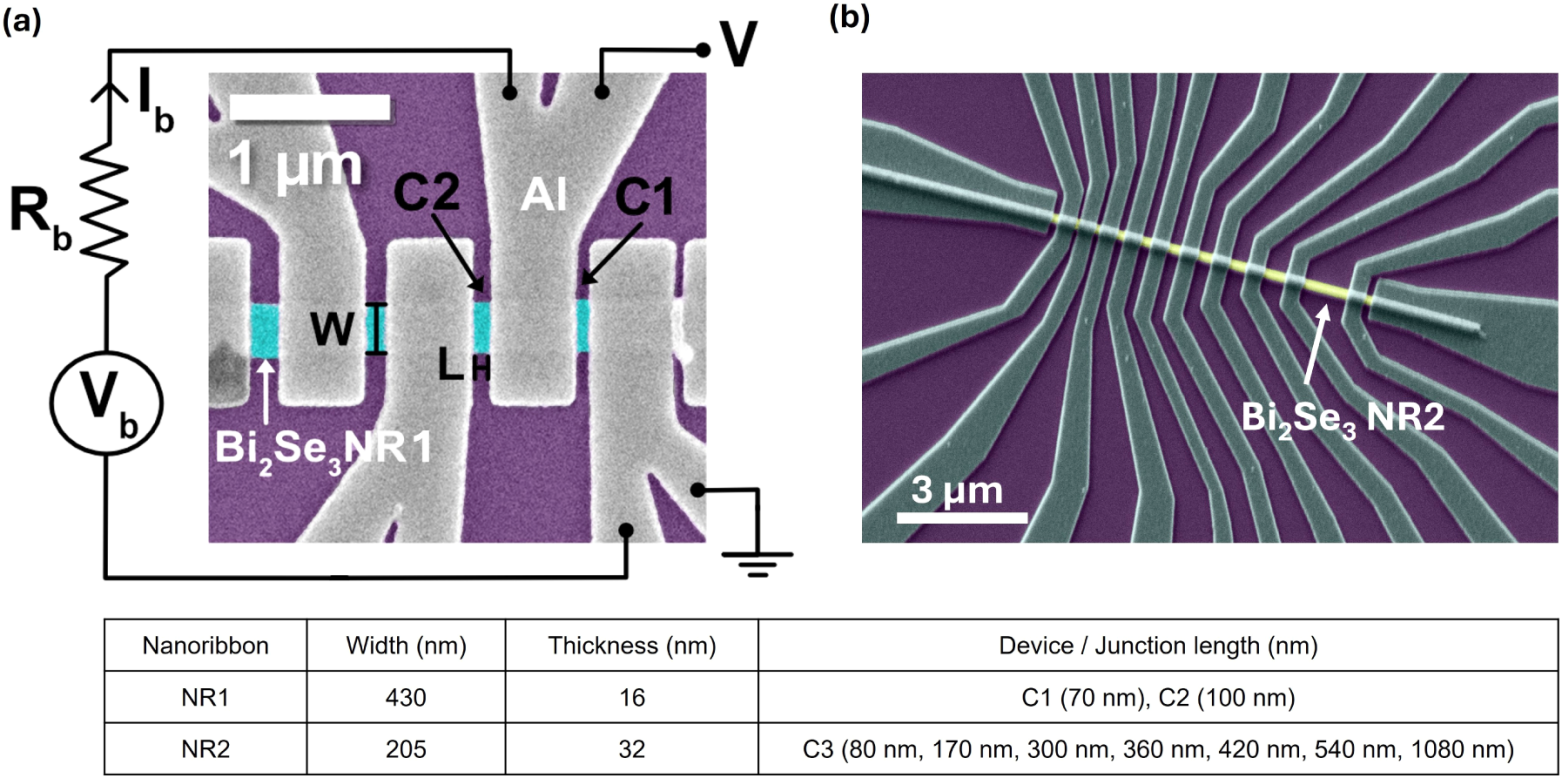
False-colored SEM images of two nanoribbons, **NR1** and **NR2**. (a) Devices **C1** and **C2** fabricated on **NR1** along with the measurement layout. (b) Josephson junctions with varying lengths, ranging from 80 nm to 1 μm, fabricated on a single Bi_2_Se_3_ nanoribbon (**NR2**). The dimensions of all measured devices are summarized in the table below.

**Figure 4:**
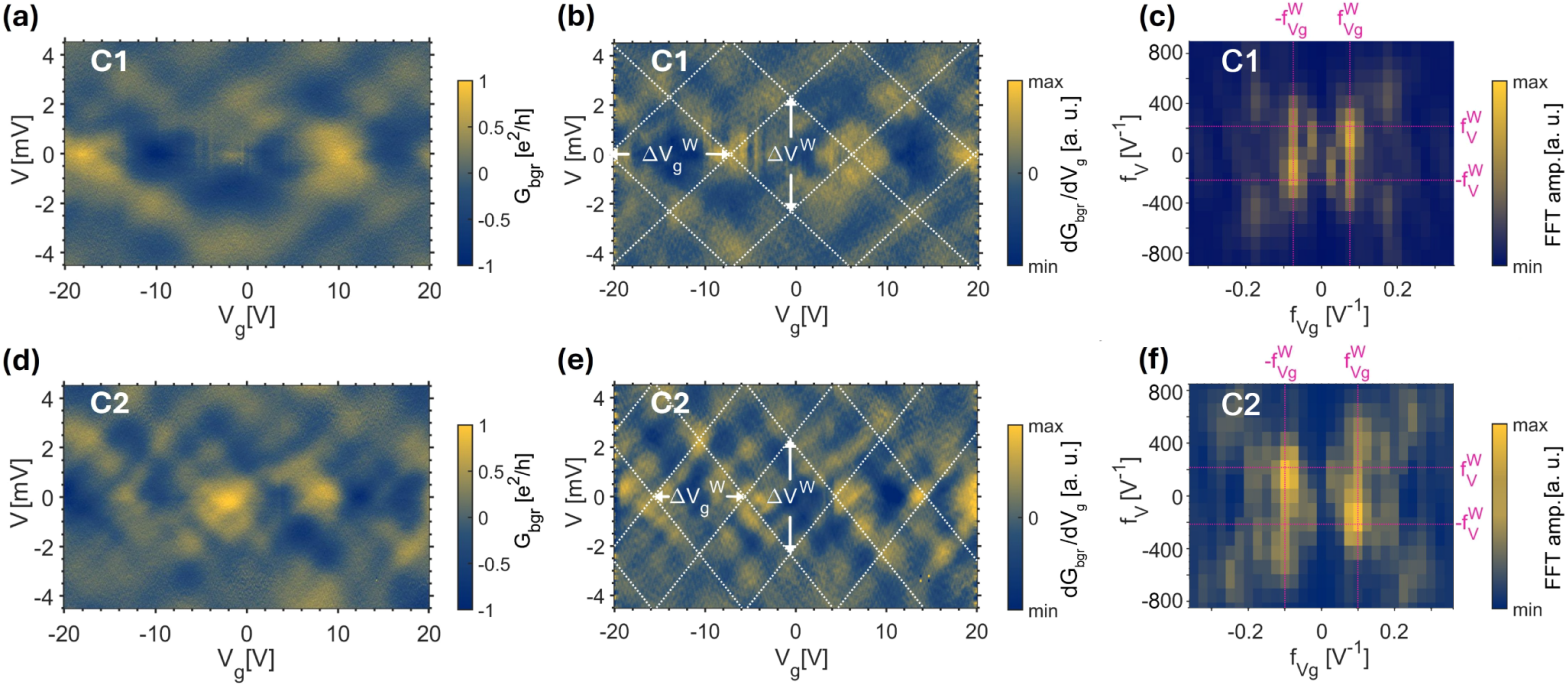
(a) Background-subtracted differential conductance map of device **C1** as a function of bias voltage *V* and back-gate voltage *V_g_*, showing Fabry–Pérot resonance patterns. (b) Numerical derivative of the conductance with respect to gate voltage *dG_bgr_*/*dV_g_*, obtained from the data in panel (a). The white dashed lines indicate the expected location of Fabry–Pérot resonances originating from quantization along the nanoribbon width. (c) 2D-FFT of the color map from panel (b), revealing four clear peaks (highlighted in orange) at $f_{V}^{W}≃\pm 204\;V^{-1}$ , corresponding to the expected frequency value of 208 V^–1^ (horizontal magenta dashed lines), consistent with transverse quantization. (d–f) The same analysis applied to device **C2** (*L* = 100 nm). The corresponding 2D-FFT again shows well-defined peaks at $f_{V}^{W}≃\pm 216\;V^{-1}$ along *f_V_*.

These results are in very close agreement with our recently published work,^37^ in which we demonstrated that crossings of sub-bands by the Fermi level (induced by the back gate) lead to pronounced peaks in the zero-bias conductance as a function of gate voltage. Due to gate leakage at elevated |*V*_*g*_| and overheating at elevated |*V*|, we could not extend the measurement window sufficiently to reach the energy scale associated with length quantization (Δμ^*L*^ = 14.8 meV). Therefore, a length-related periodicity is not discernible in our data.

The diamond-shaped patterns observed in Figures 4b and e are not perfectly sharp and may contain contributions from additional conductance fluctuations, including possible universal conductance fluctuations originating from diffusive bulk transport modes. To assess whether the observed conductance patterns are primarily governed by UCF, we performed additional measurements on the same devices at zero externally applied magnetic field.

Figure 5 shows color maps of the derivative *dG*_*bgr*_/*dV*_*g*_ as a function of bias and gate voltages for devices **C1** and **C2** on nanoribbon **NR1**, measured over a reduced gate-voltage range of ±10 V. This range is smaller than the ±20 V range presented in Figure 4 and allows finer details of the oscillation patterns to be resolved. Moreover, at zero field, the devices are in the superconducting state (Figures 5b,d), and additional subgap features appear due to multiple Andreev reflection processes within approximately ±2Δ. Outside this superconducting energy window, the diamond-shaped conductance fluctuation patterns at larger voltage scales remain essentially unchanged from those measured in the finite-field normal state. In contrast, at smaller voltage scales, we observe moderate variations in the conductance maps, which may be attributed to universal conductance fluctuations. These observations suggest that the dominant oscillatory features on the voltage scale associated with FP oscillations are not significantly modified by the magnetic field, while residual structure at finer voltage scales may partially originate from UCF-related magnetofingerprint contributions. We note that the applied magnetic field of 30 mT in our experiment is approximately half of the estimated correlation field *B*_*c*_ ∼ *h*/*eLW*, required to fully randomize the UCF pattern based on the bare device dimensions. Nevertheless, even at this field strength, one would expect substantial decorrelation of UCF, which we do not observe. The observation of Fabry–Pérot resonances associated with finite-size quantization in the transverse direction in two devices with a width of *W* = 430 nm support the interpretation that phase-coherent quasi-ballistic transport contributes significantly to the measured conductance oscillations over length scales approaching a micrometer.

**Figure 5:**
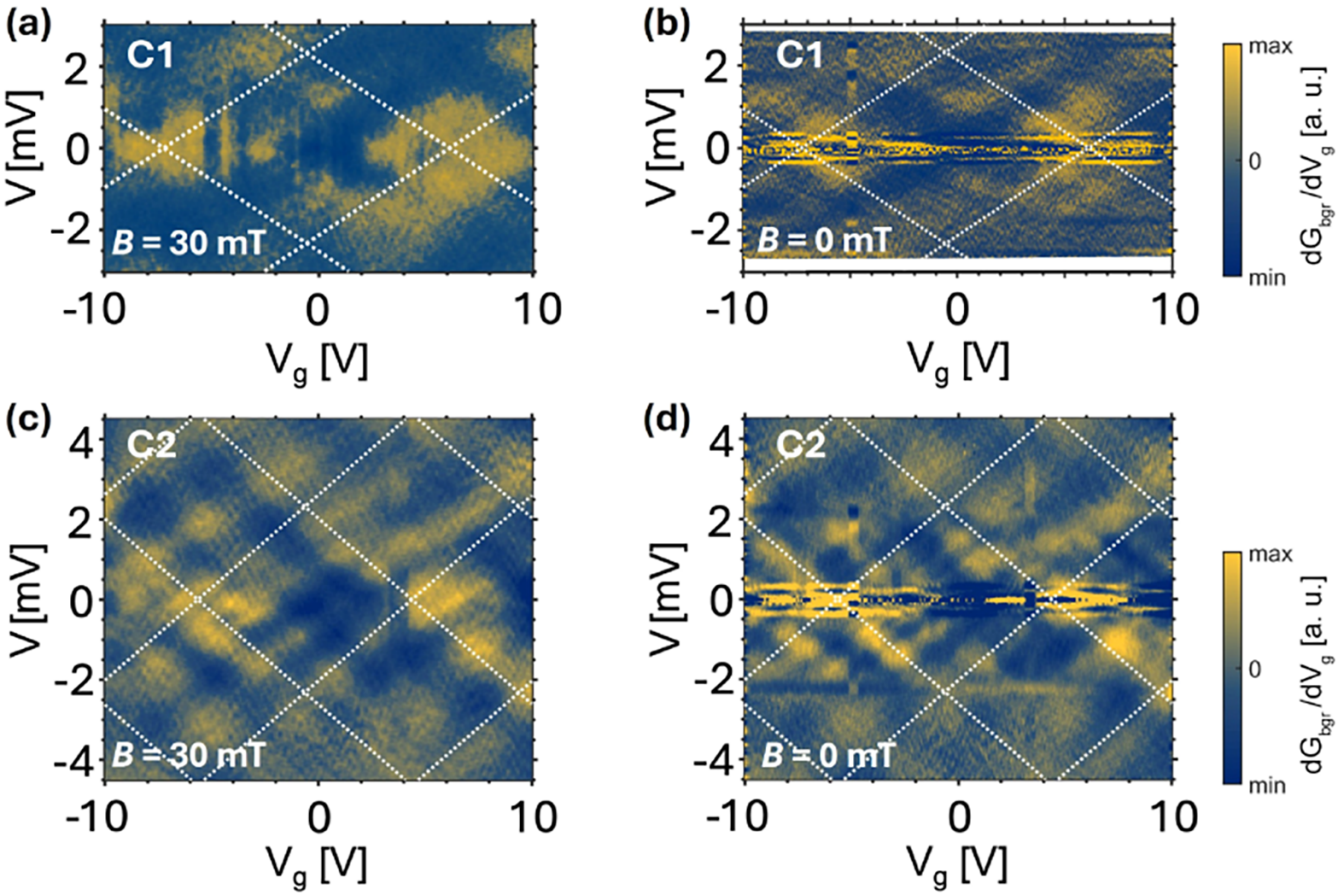
Color maps of the derivative *dG_bgr_*/*dV_g_* as a function of bias and gate voltages for device **C1**, measured in the normal state at *B_ext_* = 30 mT (a) and in the superconducting state at *B_ext_* = 0 mT (b). Panels (c) and (d) show the same measurements for device **C2**, taken in the normal state (*B_ext_* = 30 mT) and superconducting state (*B_ext_* = 0 mT), respectively.

Building on the FP evidence for quasi-ballistic coherence, we turn to the Josephson effect to probe the quasi-ballistic transport properties of the TI surface states. Previous studies on TI Josephson junctions have used the temperature dependence of the Josephson current to separate diffusive bulk and surface state contributions. Bulk currents are rapidly suppressed with increasing temperature, while surface-dominated currents persist to higher temperatures.^38^ In our devices, where critical currents are relatively small (a few tens of nA to ∼250 nA), thermally induced reduction of the switching current or rounding of the IVCs at elevated temperatures would make such an analysis cumbersome. Therefore, instead of performing *I*_*c*_(*T*) measurements, we fix the temperature at a base value of 20 mK and instead vary the junction length.

In Figure 3b, we show nanoribbon **NR2** with junctions of various lengths ranging from 80 to 1080 nm. From the conductance spectra as a function of the bias voltage, we extract an induced gap of Δ′ ∼ 159 ± 5 μeV for all junctions (see Figure S3d, Supporting Information 2). Moreover, comparing 2- and 4-point measurements of the IVCs, we infer a contact resistance of ∼1 Ω, much smaller than the overall junction resistance. Together with the observation of multiple Andreev reflection (MAR) subgap features^39^ (see Figure S3d, Supporting Information 2) for details, this indicates highly transparent interfaces between the Al electrodes and the nanoribbon.

From the measured IVCs, we first extract the normal resistance of the junctions at voltages above Δ′/*e* (see Supporting Information 2). The resulting *R*_*n*_ values are plotted as a function of junction length in Figure 6a. The resistance increases with junction length, with a steeper slope at short lengths. To qualitatively describe this behavior, we employ a phenomenological two-channel model consisting of a diffusive bulk contribution connected in parallel with a surface-state transport channel. The bulk contribution is assumed to be purely diffusive over the entire length range and is described by a linear dependence: *R*_*bulk*_(*L*) = α_*bulk*_ *L*, where *L* is the length of the junction and α_*bulk*_ is the resistance per unit length of the bulk transport channel. The surface state contribution is described by two regimes. For short junctions, we assume that resistance remains constant, *R*_*surf*_(*L*) = *R*_0_, consistent with quasi-ballistic transport. For larger junction lengths, a diffusive-like linear increase is assumed, *R*_*surf*_(*L*) = α_*surf*_ *L*, with α_*surf*_ being the resistance per unit length of the surface states, reflecting increased scattering at larger propagation lengths. We can therefore parametrize the resistance of the surface states as *R*_*surf*_(*L*) = max­(*R*_0_,α_*surf*_ *L*). The total resistance is then obtained from the parallel combination $R_{tot}^{-1}=R_{bulk}^{-1}+R_{surf}^{-1}$ . We emphasize that this model is intended as a qualitative phenomenological description of the observed length dependence rather than a unique microscopic extraction of bulk and surface transport contributions. Nevertheless, we can fit the total resistance to our measured resistance data and obtain the three fitting parameters: *R*_0_ = 743 ± 265 Ω, α_*surf*_ = 1.56 ± 0.37 Ω/nm, and α_*bulk*_ = 3.63 ± 1.87 Ω/nm (Figure 6a).

**Figure 6:**
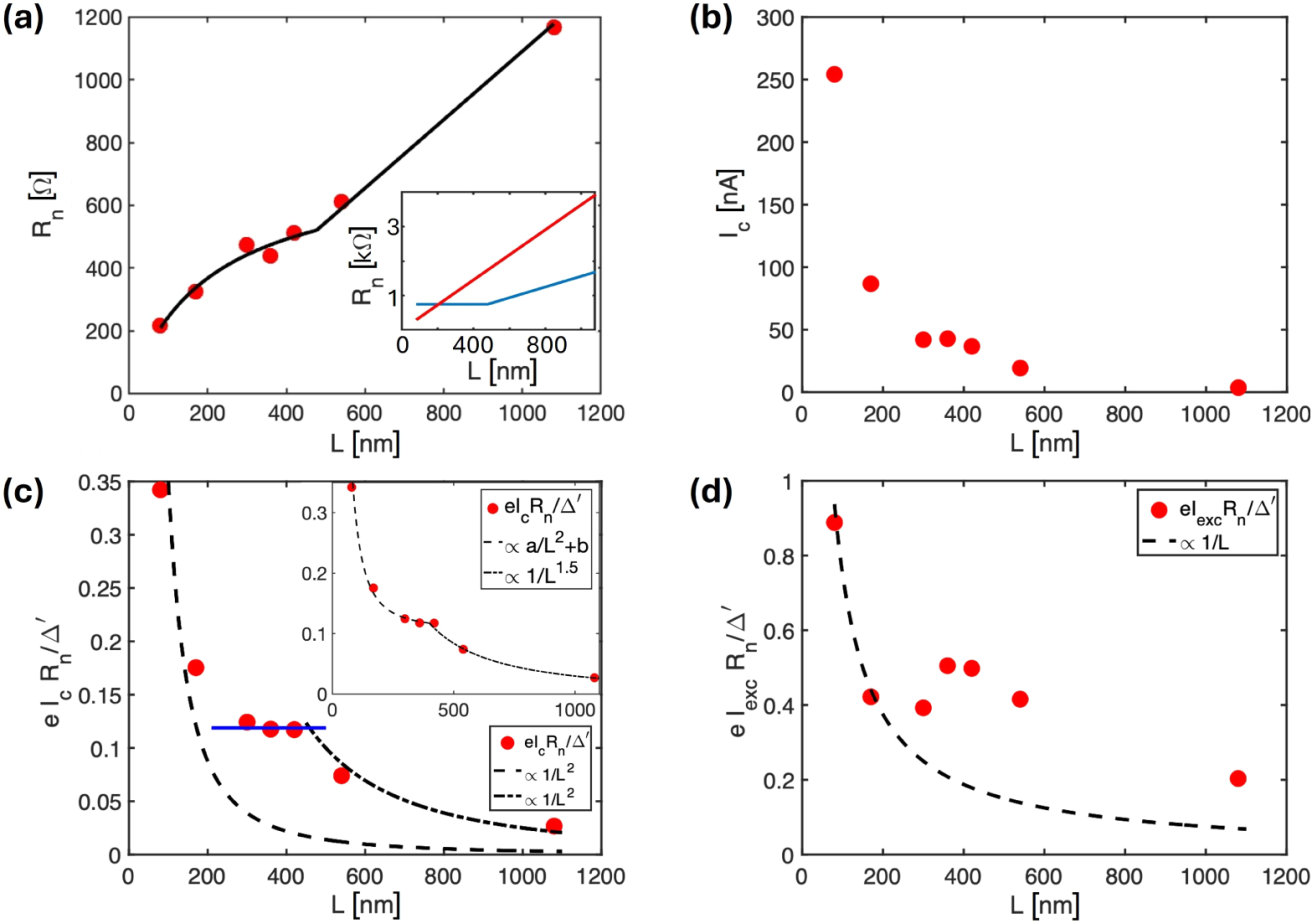
Length dependence of transport parameters for **NR2** Bi_2_Se_3_ nanoribbon Josephson junctions. (a) Normal-state resistance *R_n_* as a function of junction length (red dots). The solid line shows a fit to two parallel transport channels (shown in the inset): a diffusive bulk contribution (red) and surface state conduction (blue). (b) Critical current *I_c_* vs junction length. (c) *I_c_R_n_* product, normalized to Δ′/*e*, as a function of junction length, *L*. The dashed and dash-dotted curves illustrate the 1/*L* ^2^ dependence expected for long diffusive junctions. Inset: alternative crossover representation of the same data. The dashed line shows a fit using *a*/*L* ^2^ + *b* for *L* < 500 nm, while the dash-dotted line illustrates a fit to a *a*_surf_/*L* ^1.5^ for longer junctions. (d) Excess current, normalized to Δ′/*R_ne_*, as a function of junction length. The dashed line illustrates the 1/*L* dependence expected for long diffusive junctions.

In Figure 6b, we show the critical currents *I*_*c*_(*L*) extracted from the IVCs as a function of junction length (Figure S3 (a,b)). The critical current decreases rapidly for *L* ≤ 200 nm, exhibits a plateau between 300 and 420 nm, and decreases again at larger *L*.

The product *I*_*c*_ *R*_*n*_, normalized to Δ′/*e* using the data from panels (a) and (b), is shown in Figure 6c. In long diffusive Josephson junctions (*L* > *ξ* ^diff^), the characteristic voltage is expected to scale with the Thouless energy *E*_*Th*_ ∝ 1/*L* ^2^, resulting in *I*_*c*_ *R*_*n*_ ∝ 1/*L* ^2^. In contrast, for short ballistic junctions (*L* < *ξ* ^b^), the *I*_*c*_ *R*_*n*_ product remains approximately constant and proportional to the induced superconducting gap Δ′.^40^ Using carrier densities and mobilities previously extracted from magnetotransport measurements on Bi_2_Se_3_ nanoribbons in our earlier studies,^33–35^ we estimate electron mean free paths of ≈20–40 nm for the bulk states and ≈130–150 nm for the topological surface states. These values correspond to diffusive coherence lengths ξ^diff^ of approximately 115–160 nm for the bulk channel and ≈365 nm for the TSS contribution, while the ballistic coherence length is estimated as ξ^b^ = ℏ*v*_*F*_ /Δ′ ≈ 2 μm (see Supporting Information 4).

The initial decrease of the *I*_*c*_ *R*_*n*_ product approximately follows a 1/*L* ^2^ dependence, consistent with diffusive bulk transport. At larger junction lengths, a plateau-like region emerges and extends up to *L* ≃ 500 nm (solid blue line), where the diffusive bulk contribution is strongly suppressed (dashed line), while phase-coherent transport through the surface state channel remains in the short ballistic regime. At even larger junction lengths, the *I*_*c*_ *R*_*n*_ product associated with surface states begins to decay (dash-dotted line), indicating that some transport trajectories become diffusive, although ballistic paths may still persist.

The same data can also be described within a crossover picture, shown in the inset of Figure 6c. For junction lengths below ∼500 nm, the data are well described by the expression *a*/*L* ^2^ + *b*, with *a* = 1700 nm^2^ and *b* = 17. Here, the 1/*L* ^2^ term is attributed to the diffusive bulk contribution, while the constant offset *b* reflects a quasi-ballistic surface state contribution. For larger junction lengths, the plateau evolves into a power-law decay that can be approximated by *a*_surf_/*L* ^1.5^, with *a*_surf_ = 940  nm^1.5^, indicating a gradual crossover from short-ballistic to increasingly diffusive transport within the surface state channel. The exponent smaller than 2 suggests that junctions in this regime may not yet be fully in the long-junction limit, i.e., *L ∼ ξ* rather than *L* ≫ *ξ*. This crossover description is fully consistent with the phenomenological interpretation discussed above and further supports the picture of a gradual evolution from quasi-ballistic to diffusive transport with increasing junction length.

To corroborate this behavior, we also plot the excess current, normalized to Δ′/*R*_*n*_ *e*, which reflects the overall junction transparency.^41^ In contrast to the 1/*L* scaling expected for long diffusive junctions,^42^ we observe a saturation over length scales of ∼300–500 nm. Interestingly, the mean free path extracted from conventional magnetotransport measurements in our nanoribbons is only on the order of ∼150 nm for typical mobility values of ∼4000 cm^2^/(V·s),^33,35^ representing an average over both ballistic and diffusive transport modes. These observations suggest that Josephson and FP interference measurements are considerably more sensitive probes of ballistic transport modes than conventional magnetotransport techniques.

## Conclusions

Our measurements show that Bi_2_Se_3_ nanoribbon Josephson junctions host phase-coherent surface state trajectories that support Fabry–Pérot oscillations associated with transverse quantization in 430 nm wide devices. In a complementary junction-length series spanning submicrometer channel lengths, the normal-state resistance is well described by a parallel combination of a diffusive bulk channel and a surface state contribution, while the Josephson characteristic voltage *I*_*c*_ *R*_*n*_ remains nearly constant up to *L* ≃ 500 nm after an initial decay, and subsequently decreases at larger lengths. Taken together, these results demonstrate substantial quasi-ballistic surface state transport over several hundred nanometers in topological insulator nanostructures, coexisting with diffusive bulk conduction. The combined use of Fabry–Pérot spectroscopy and Josephson transport provides a sensitive, channel-selective approach for probing coherent quantum transport in nanoscale topological insulator devices. These findings establish Bi_2_Se_3_ nanoribbon Josephson junctions as a promising platform for phase-coherent superconducting electronics and offer guidance for engineering superconducting TI devices, where long phase-coherent paths are desirable.

## Supplementary Material



## Data Availability

Data sets generated during the current study are available from the corresponding author on reasonable request.

## Notes

- 1. Electrochemical potential diagrams for symmetric and asymmetric biasing configurations; calculated finite-bias Fabry–Pérot conductance maps and corresponding 2D-FFT analysis for device dimensions comparable to the experimental junctions.
- 2. Representative current–voltage characteristics and differential-resistance measurements; extraction of critical current, normal-state resistance, excess current, and induced superconducting gap.
- 3. Raw normal-state differential conductance maps for devices **C1** and **C2** before background subtraction.
- 4. Estimation of electron mean free paths, superconducting coherence lengths, and expected *I_c_R_n_* scaling in ballistic and diffusive transport regimes (PDF)

## References

[1] Fu L., Kane C. L. (2008). Superconducting Proximity Effect and Majorana Fermions
at the Surface of a Topological Insulator. Phys.
Rev. Lett..

[2] Nayak C., Simon S. H., Stern A., Freedman M., Das Sarma S. (2008). Non-Abelian
Anyons and Topological Quantum Computation. Rev. Mod. Phys..

[3] Hasan M. Z., Kane C. L. (2010). Colloquium: Topological Insulators. Rev. Mod. Phys..

[4] Dankert A., Geurs J., Kamalakar M. V., Charpentier S., Dash S. P. (2015). Room Temperature Electrical Detection
of Spin Polarized
Currents in Topological Insulators. Nano Lett..

[5] He M., Sun H., He Q. L. (2019). Topological
Insulator: Spintronics and Quantum Computations. Front. Phys..

[6] Breunig O., Ando Y. (2022). Opportunities in Topological
Insulator Devices. Nat. Rev. Phys..

[7] Rittenhouse G. E., Graybeal J. M. (1994). Fabry-Perot Interference
Peaks in the Critical Current
for Ballistic Superconductor-Normal-Metal-Superconductor Josephson
Junctions. Phys. Rev. B.

[8] Sahu S. K., Soori A. (2023). Fabry-Perot Interference
in Josephson Junctions. Eur. Phys. J. B.

[9] Liang W., Bockrath M., Bozovic D., Hafner J. H., Tinkham M., Park H. (2001). Fabry-Perot Interference
in a Nanotube Electron Waveguide. Nature.

[10] Miao F., Wijeratne S., Zhang Y., Coskun U. C., Bao W., Lau C. N. (2007). Phase-Coherent
Transport in Graphene Quantum Billiards. Science.

[11] Young A. F., Kim P. (2009). Quantum Interference
and Klein Tunnelling in Graphene Heterojunctions. Nat. Phys..

[12] Kretinin A.
V., Popovitz-Biro R., Mahalu D., Shtrikman H. (2010). Multimode
Fabry-Pérot Conductance Oscillations in Suspended Stacking-Faults-Free
InAs Nanowires. Nano Lett..

[13] Grushina A. L., Ki D. K., Morpurgo A. F. (2013). A Ballistic p–n Junction in
Suspended Graphene with Split Bottom Gates. Appl. Phys. Lett..

[14] Rickhaus P., Maurand R., Liu M. H., Weiss M., Richter K., Schönenberger C. (2013). Ballistic
Interferences in Suspended Graphene. Nat. Commun..

[15] Varlet A., Liu M.-H., Krueckl V., Bischoff D., Simonet P., Watanabe K., Taniguchi T., Richter K., Ensslin K., Ihn T. (2014). Fabry-Pérot
Interference in Gapped Bilayer Graphene with Broken
Anti-Klein Tunneling. Phys. Rev. Lett..

[16] Rickhaus P., Makk P., Liu M. H., Tóvári E., Weiss M., Maurand R., Richter K., Schönenberger C. (2015). Snake Trajectories
in Ultraclean Graphene p–n Junctions. Nat. Commun..

[17] Pandey P., Kraft R., Krupke R., Beckmann D., Danneau R. (2019). Andreev Reflection
in Ballistic Normal Metal/Graphene/Superconductor Junctions. Phys. Rev. B.

[18] Finck A. D., Kurter C., Hor Y. S., Van Harlingen D. J. (2014). Phase Coherence
and Andreev Reflection in Topological Insulator Devices. Phys. Rev..

[19] Finck A. D., Kurter C., Huemiller E. D., Hor Y. S., Van Harlingen D. J. (2016). Robust
Fabry-Perot Interference in Dual-Gated Bi_2_Se_3_ Devices. Appl. Phys. Lett..

[20] Karalic M., Štrkalj A., Masseroni M., Chen W., Mittag C., Tschirky T., Wegscheider W., Ihn T., Ensslin K., Zilberberg O. (2020). Electron-Hole Interference in an Inverted-Band Semiconductor
Bilayer. Phys. Rev. X.

[21] Katsnelson M. I., Novoselov K. S., Geim A. K. (2006). Chiral Tunnelling and the Klein Paradox
in Graphene. Nat. Phys..

[22] Beenakker C. W. J. (2008). Colloquium:
Andreev Reflection and Klein Tunneling in Graphene. Rev. Mod. Phys..

[23] Shytov A. V., Rudner M. S., Levitov L. S. (2008). Klein Backscattering
and Fabry-Pérot
Interference in Graphene Heterojunctions. Phys.
Rev. Lett..

[24] Müller M., Bräuninger M., Trauzettel B. (2009). Temperature Dependence of the Conductivity
of Ballistic Graphene. Phys. Rev. Lett..

[25] Osca J., Moors K., Sorée B., Serra L. (2021). Fabry-Pérot
Interferometry with Gate Tunable 3D Topological Insulator Nanowires. Nanotechnology.

[26] Gunlycke D., White C. T. (2008). Graphene Interferometer. Appl.
Phys. Lett..

[27] Oksanen M., Uppstu A., Laitinen A., Cox D. J., Craciun M. F., Russo S., Harju A., Hakonen P. (2014). Single-mode and Multimode
Fabry-Pérot Interference in Suspended Graphene. Phys. Rev. B.

[28] Cook A. M., Vazifeh M. M., Franz M. (2012). Stability of Majorana Fermions in
Proximity-coupled Topological Insulator Nanowires. Phys. Rev. B.

[29] Jauregui L. A., Pettes M. T., Rokhinson L. P., Shi L., Chen Y. P. (2016). Magnetic
Field-induced Helical Mode and Topological Transitions in a Topological
Insulator Nanoribbon. Nat. Nanotechnol..

[30] Titov M., Beenakker C. W. J. (2006). Josephson Effect in Ballistic Graphene. Phys. Rev. B.

[31] Andzane J., Kunakova G., Charpentier S., Hrkac V., Kienle L., Baitimirova M., Bauch T., Lombardi F., Erts D. (2015). Catalyst-free
Vapour-Solid Technique for Deposition of Bi_2_Te_3_ and Bi_2_Se_3_ Nanowires/Nanobelts with Topological
Insulator Properties. Nanoscale.

[32] Münning F., Breunig O., Legg H. F., Roitsch S., Fan D., Rößler M., Rosch A., Ando Y. (2021). Quantum Confinement
of the Dirac Surface States in Topological-Insulator Nanowires. Nat. Commun..

[33] Kunakova G., Galletti L., Charpentier S., Andzane J., Erts D., Léonard F., Spataru C. D., Bauch T., Lombardi F. (2018). Bulk-free
Topological Insulator Bi_2_Se_3_ Nanoribbons with
Magnetotransport Signatures of Dirac Surface States. Nanoscale.

[34] Sondors R., Niherysh K., Andzane J., Palermo X., Bauch T., Lombardi F., Erts D. (2023). Low-Vacuum Catalyst- Free Physical
Vapor Deposition and Magnetotransport Properties of Ultrathin Bi_2_Se_3_ Nanoribbons. Nanomaterials.

[35] Kunakova G., Bauch T., Palermo X., Salvato M., Andzane J., Erts D., Lombardi F. (2021). High-Mobility Ambipolar Magnetotransport
in Topological Insulator Bi_2_Se_3_ Nanoribbons. Phys. Rev. Appl..

[36] Ziegler J., Kozlovsky R., Gorini C., Liu M.-H., Weishäupl S., Maier H., Fischer R., Kozlov D. A., Kvon Z. D., Mikhailov N., Dvoretsky S. A., Richter K., Weiss D. (2018). Probing Spin
Helical Surface States in Topological HgTe Nanowires. Phys. Rev. B.

[37] Niherysh K., Palermo X., Surendran A. P., Kalaboukhov A., Sondors R., Andzane J., Erts D., Bauch T., Lombardi F. (2025). Quantum Confinement and Coherent Transport in Ultrathin
Bi_2_Se_3_ Nanoribbons. Sci.
Rep..

[38] Schüffelgen P., Rosenbach D., Li C., Schmitt T. W., Schleenvoigt M., Jalil A. R., Schmitt S., Kölzer J., Wang M., Bennemann B., Parlak U., Kibkalo L., Trellenkamp S., Grap T., Meertens D., Luysberg M., Mussler G., Berenschot E., Tas N., Golubov A. A., Brinkman A., Schäpers T., Grützmacher D. (2019). Selective
Area Growth and Stencil Lithography for In-situ Fabricated Quantum
Devices. Nat. Nanotechnol..

[39] Kunakova G., Bauch T., Trabaldo E., Andzane J., Erts D., Lombardi F. (2019). High-transparency Bi_2_Se_3_ Topological
Insulator Nanoribbon Josephson Junctions with Low Resistive Noise
Properties. Appl. Phys. Lett..

[40] Dubos P., Courtois H., Pannetier B., Wilhelm F. K., Zaikin A. D., Schön G. (2001). Josephson
Critical Current in a Long Mesoscopic S-N-S
Junction. Phys. Rev. B.

[41] Flensberg K., Hansen J. B., Octavio M. (1988). Subharmonic Energy-gap
Structure
in Superconducting Weak Links. Phys. Rev. B.

[42] Cuevas J., Hammer J., Kopu J., Viljas J., Eschrig M. (2006). Proximity
Effect and Multiple Andreev Reflections in Diffusive Superconductor-Normal-metal-Superconductor
Junctions. Phys. Rev. B.

