# Supplementary material for: Al–Bi2Se3–Al Nanoribbon Josephson Junctions with Fabry–Pérot Interference: Implications for Phase-Coherent Topological Insulator-Based Superconducting Devices
Source: ACS Appl Nano Mater. 2026 Jul 1;9(27):12816–24. doi: 10.1021/acsanm.6c01243 (PMC13366489; doi:10.1021/acsanm.6c01243)
Supplement: Supplementary file 1 [file an6c01243_si_001.pdf]

Supporting Information

Al-Bi_2Se_3-Al Nanoribbon Josephson Junctions with Fabry-Pérot Interference: Implications for Phase-Coherent Topological Insulator-Based Superconducting Devices.

Kiryl Niherysh[1], **Nermin Trnjanin,**[1], **Ananthu P. Surendran**[1], **Gunta Kunakova**[1,2], **Xavier Palermo**[1], **Domenico Montemurro**[1,3], **Jana Andzane**[2], **Donats Erts**[2], **Dmitry S. Golubev**[4], **Samuel Lara-Avila**[1], **Floriana Lombardi**[1], **and Thilo Bauch**[1,*]

[1]Quantum Device Physics Laboratory, Department of Microtechnology and Nanoscience, Chalmers University of Technology, Göteborg, SE-41296, Sweden
[2]Institute of Chemical Physics, Faculty of Science and Technology, University of Latvia, Riga, LV-1586, Latvia
[3]Dipartimento di Fisica "Ettore Pancini", Università degli Studi di Napoli Federico II, Napoli, I-80125, Italy
[4]QTF Centre of Excellence, Department of Applied Physics, Aalto University, Aalto, FI-00076, Finland
[*]thilo.bauch@chalmers.se

SUPPLEMENTARY INFORMATION 1

Effect of Source-Drain Bias on the Electrochemical Potentials

To calculate the finite-bias conductance maps shown in Fig. 1(d) in the main text, we employ the Landauer formalism (Eq. 2 in the main text), in which the current is determined by the transmission probability integrated over the energy window defined by the electrochemical potentials of the two contacts: $\varepsilon_l = \mu_0 + \alpha eV$ and $\varepsilon_r = \mu_0 - (1-\alpha)eV$, with μ_0 the chemical potential measured relative to the Dirac point of the topological surface states, and α describes how the voltage drop is distributed across the junction and allows different biasing configurations to be considered (Fig. S1). For a symmetric voltage drop $\alpha = 1/2$, the two electrochemical potentials move equally in opposite directions with increasing bias $\varepsilon_l = \mu_0 + eV/2$ and $\varepsilon_r = \mu_0 - eV/2$ (see Fig. S1(b)). In this case the center of the integration window (quasi-Fermi level) remains fixed at μ_0, and the source-drain bias only increases the width of the energy window.

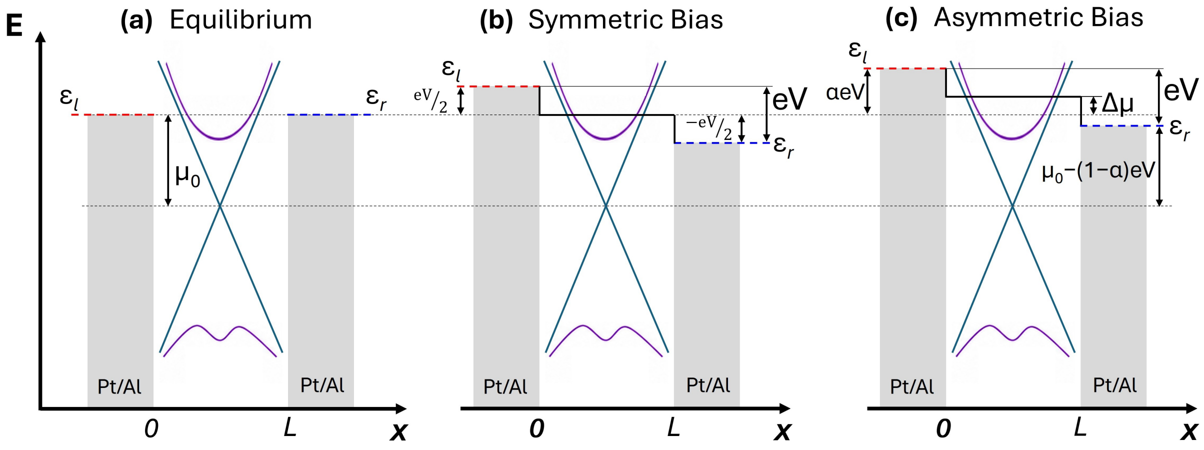

Figure S1. Schematic illustration of the electrochemical potentials in the electrode/Bi_2Se_3/electrode device relative to the Dirac point. (a) Equilibrium configuration ($V = 0$), where the electrochemical potentials of the left and right contacts are equal $\varepsilon_l = \varepsilon_r = \mu_0$. (b) Symmetric bias configuration ($\alpha = 1/2$), for which the applied source-drain voltage shifts the contact electrochemical potentials by $\pm\, eV/2$, resulting in an energy window of width eV centered at μ_0. (c) Asymmetric bias configuration ($\alpha \neq 1/2$), where the voltage drop is unequally distributed between the two interfaces. In this case, the center of the transport window is shifted relative to the Dirac point by $\Delta\mu = (\alpha - 1/2)eV$. The Dirac point remains fixed, while the electrochemical potentials move with respect to it.

More generally, the center of the bias window is located at $(\varepsilon_l + \varepsilon_r)/2 = \mu_0 + (\alpha - 1/2)eV$. Therefore, for asymmetric bias configurations ($\alpha \neq 1/2$), the center of the integration window shifts linearly with applied bias relative to the Dirac point $\Delta\mu = (\alpha - 1/2)eV$ (see Fig. S1(c)). The Dirac point itself remains fixed, rather the electrochemical potentials move with respect to it. This shift modifies the energies sampled by transport and can alter the detailed appearance of the finite-bias conductance maps.

The calculations presented in the main text assume symmetric biasing ($\alpha = 1/2$), for which the electrochemical potentials of the two contacts move equally in opposite directions with increasing source-drain voltage. As a result, resonant transmission occurs whenever either edge of the bias window crosses a Fabry-Pérot resonance, producing two equivalent resonance branches with opposite slopes in the (V, μ)-plane. Their intersection gives rise to the characteristic diamond-shaped interference pattern. Under these conditions, the FP periodicities associated with quantization along the junction length and width appear in the finite-bias conductance map as $\Delta V^L = 2\Delta\mu^L/e$, and $\Delta V^W = 2\Delta\mu^W/e$, respectively. The factor of two arises because the applied bias changes the energies of the two contacts by $\pm\, eV/2$, so that the total width of the transport window is eV.

Here, we additionally present calculated differential conductance map and the corresponding 2D Fourier transform (symmetric biasing configuration) for a device with dimensions $L = 100$ nm and $W = 500$ nm, similar to devices **C1** and **C2** discussed in the main text (Fig. S2). We note that the Fabry-Pérot peaks in the simulated 2D-FFT maps are relatively broad rather than perfectly sharp.

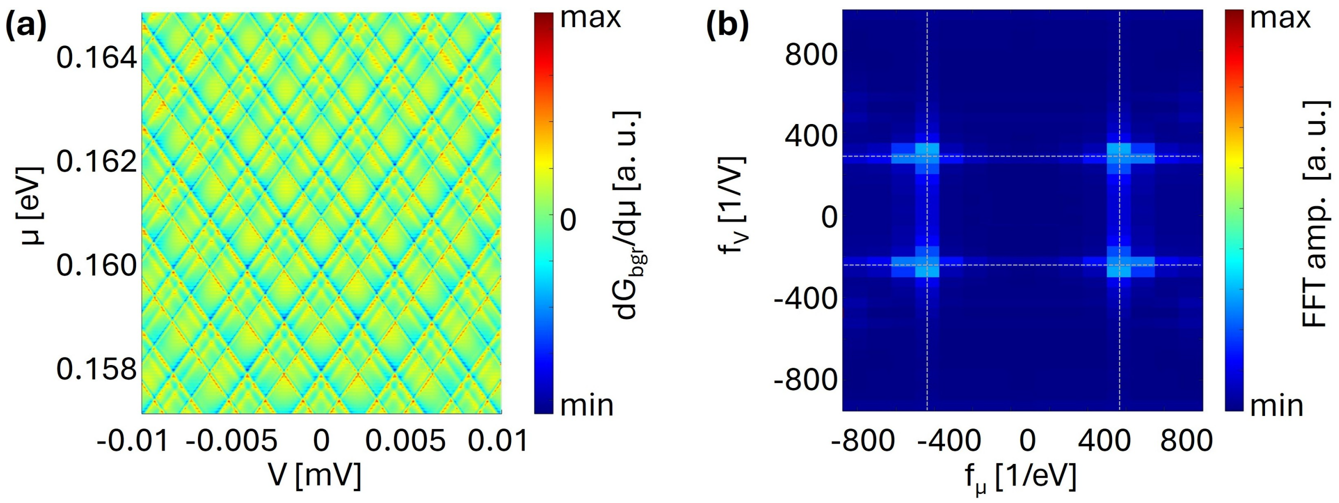

Figure S2. (a) $dG_{bgr}/d\mu$ as a function of source-drain voltage V and chemical potential μ for a device with $L = 100$ nm and $W = 500$ nm, calculated for symmetric biasing ($\alpha = 1/2$). (b) The corresponding 2DFFT of the conductance map from panel (a). Dashed lines are guides to the eye indicating the displacement of the dominant FFT peaks.

SUPPLEMENTARY INFORMATION 2

Extraction of Transport Parameters from IV Characteristics

Fig. S3 shows representative transport characteristics of the Josephson junctions on nanoribbon **NR2** ($T = 20$ mK). The low-bias current-voltage characteristic (IVC) for a 300 nm-long junction, shown in panel (a), exhibits a clear transition from the superconducting to the resistive state. The corresponding differential resistance dV/dI in panel (b) reveals distinct extrema, where the outer peaks define the switching currents (I_c^+ and I_c^-), while the inner features are associated with retrapping processes. At higher bias (panel (c)), the IVC becomes linear. This allows to determine the normal-state resistance R_n and the excess current I_{exc} by fitting the normal branch with a straight line. The excess current is obtained by extrapolating the linear fit to zero voltage, while the resistance is given by the inverse of the slope of the linear fit. The fitting is performed within a voltage window $350\,\mu\text{V} < |V| < 450\,\mu\text{V}$ to avoid heating effects at larger bias. Panel (d) shows the differential resistance as a function of bias voltage for junctions of different lengths (**NR2**). Pronounced subharmonic gap features are observed, consistent with multiple Andreev reflection processes. From their positions, we extract an induced superconducting gap of $\Delta' \simeq 159 \pm 5\ \mu$eV, which is consistent across all devices.

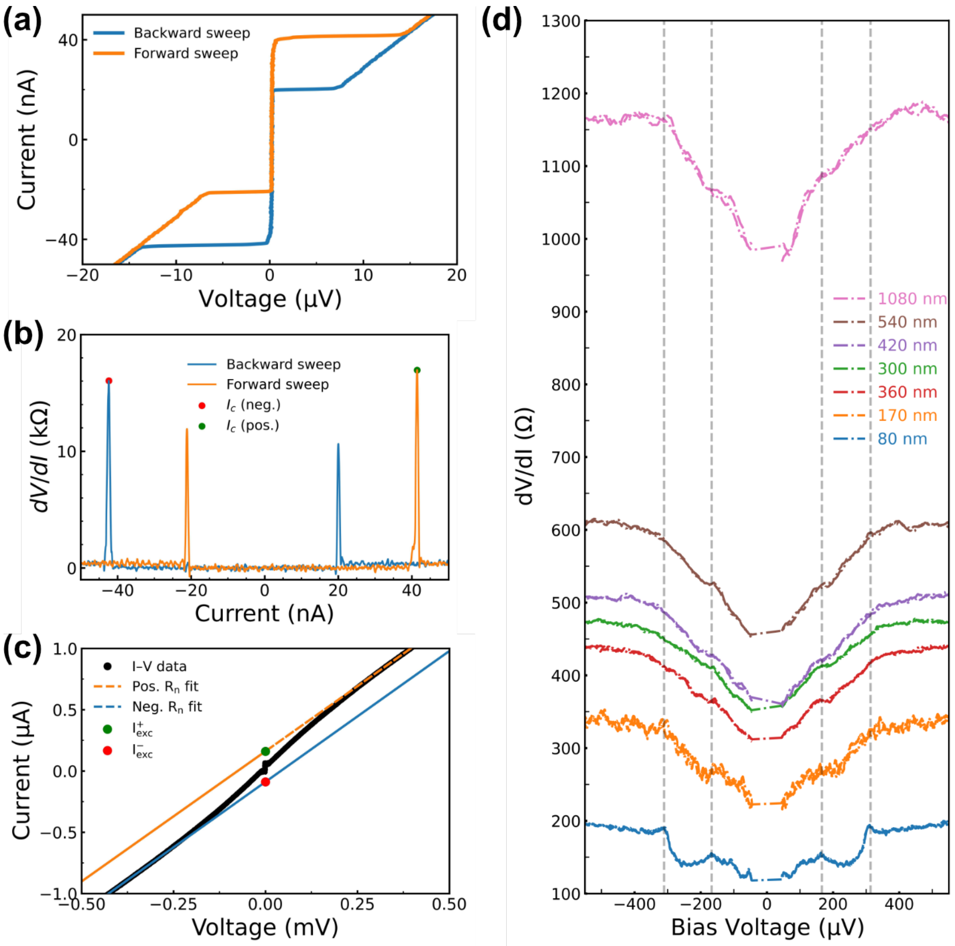

Figure S3. Transport characterization of representative Josephson junctions on nanoribbon **NR2** ($T = 20$ mK). (a) Low-bias current-voltage characteristic (IVC) of a 300 nm junction, showing the switching from the superconducting to the resistive state. (b) Differential resistance dV/dI as a function of bias current, used to extract the critical current I_c. (c) High-bias IVC used to determine the normal-state resistance R_n and excess current I_{exc} via linear fits to the normal branch. (d) Differential resistance vs. bias voltage for junctions of different lengths (**NR2**). Dashed lines mark the positions of the induced gap Δ'/e and $2\Delta'/e$. Note that the central low-bias region around zero voltage was removed for clarity, and the positive- and negative-bias branches are connected graphically.

SUPPLEMENTARY INFORMATION 3

Raw conductance maps

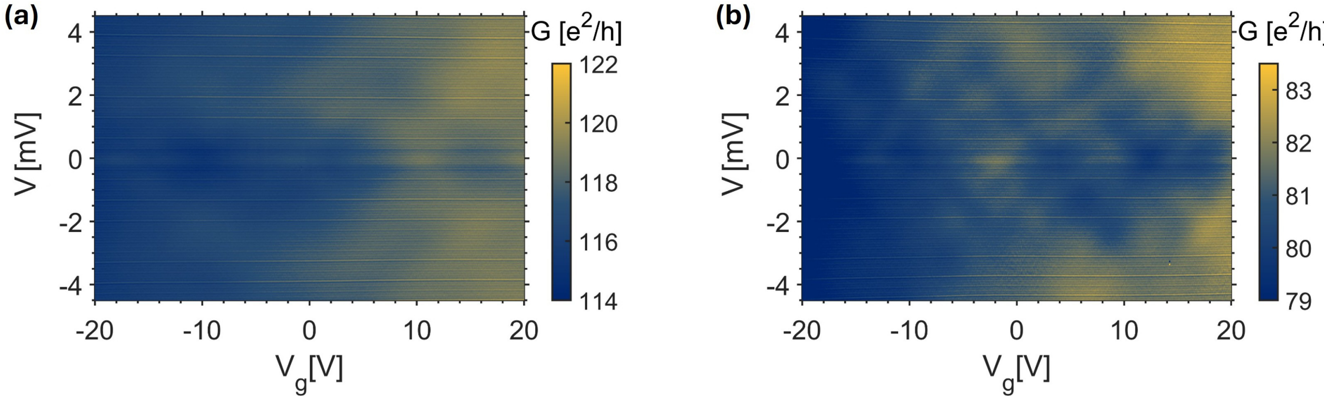

Figure S4. Raw differential conductance maps in the normal state for devices **C1** (a) and **C2** (b), respectively.

The raw differential conductance maps in the normal state, without background subtraction, are shown in Fig. S4(a,b) for devices **C1** and **C2**, respectively. The oscillatory patterns observed in the raw maps are consistent with those revealed more clearly in the background-subtracted data shown in Fig.3(b) and (e). This agreement demonstrates that the observed Fabry-Pérot-type features are intrinsic to the devices rather than artifacts introduced by the data-processing procedure.

SUPPLEMENTARY INFORMATION 4

I_cR_n product and characteristic length scales

Mean free path: For the topological surface states (TSS), the electronic mean free path is given by $l_e^{TSS} = \mu\hbar\sqrt{4\pi n_{2D}}/e$, where μ is the carrier mobility, $\hbar$ is the reduced Planck constant, n_{2D} is the 2D carrier density, and e is the elementary charge. For bulk states, the mean free path is $l_e^{bulk} = \mu m^* v_F/e$, where $m^* \sim 0.15 m_e$ is the effective mass, m_e is the free-electron mass, and $v_F \sim 5 \cdot 10^5$ m/s is the Fermi velocity for a chemical potential close to the edge of the conduction band[1,2].

I_cR_n **product:** For a long diffusive Josephson junction $L > \xi^{diff}$ in the zero-temperature limit, the I_cR_n product is proportional to the Thouless energy $\hbar D/L^2$, where D is the diffusion constant[3]. The superconducting coherence length in the diffusive limit is given by $\xi^{diff} = \sqrt{\hbar D/\Delta'}$ with the induced superconducting gap $\Delta' \sim 160$ μeV[4]. The diffusion constant is $D = v_F l_e/2$ for a 2D system and $D = v_F l_e/3$ for a 3D system. For a short ballistic Josephson junction, $L < \xi^b$, the I_cR_n product is constant and proportional to the induced gap Δ'. Here, $\xi^b = \hbar v_F/\Delta' \simeq 2$ μm is the superconducting coherence length in the ballistic limit.

References

1. Kunakova, G., Galletti, L., Charpentier, S., Andzane, J., Erts, D., Léonard, F., Spataru, C. D., Bauch, T. & Lombardi, F. Bulk-free Topological Insulator Bi_2Se_3 Nanoribbons with Magnetotransport Signatures of Dirac Surface States. *Nanoscale* **10**, 19595–19602, DOI: https://doi.org/10.1039/c8nr05500a (2018).

2. Kunakova, G., Bauch, T., Palermo, X., Salvato, M., Andzane, J., Erts, D. & Lombardi, F. High-Mobility Ambipolar Magnetotransport in Topological Insulator Bi_2Se_3 Nanoribbons. *Phys. Rev. Appl.* **16**, 024038, DOI: https://doi.org/10.1103/PhysRevApplied.16.024038 (2021).

3. Zaikin, A. & Zharkov, G. Effect of External Fields and Impurities on the Josephson Current in SNINS Junctions. *Zh. Eksp. Teor. Fiz.* **81**, 1781–1802 (1981).

4. Kunakova, G., Bauch, T., Trabaldo, E., Andzane, J., Erts, D. & Lombardi, F. High-transparency Bi_2Se_3 Topological Insulator Nanoribbon Josephson Junctions with Low Resistive Noise Properties. *Appl. Phys. Lett.* **115**, 172601, DOI: https://doi.org/10.1063/1.5123554 (2019).
